# Dementia and the Paradigm of the Camp: Thinking Beyond Giorgio Agamben’s Concept of “Bare Life”

**DOI:** 10.1007/s11673-019-09913-5

**Published:** 2019-05-27

**Authors:** Lucy Burke

**Affiliations:** Department of English, Faculty of Arts and Humanities, Righton Building, Manchester Metropolitan University, All Saints South Campus, Manchester, M15 6LL UK

**Keywords:** Dementia, Literature and life-writing, Personhood, Human value, Care, Holocaust, Giorgio Agamben, Bare life

## Abstract

This essay discusses the use of analogies drawn from the Holocaust in cultural representations and critical scholarship on dementia. The paper starts with a discussion of references to the death camp in cultural narratives about dementia, specifically Annie Ernaux’s account of her mother’s dementia in *I Remain in Darkness*. It goes on to develop a critique of Italian philosopher Giorgio Agamben’s work on biopolitics and “bare life,” focusing specifically on the linguistic foundations of his thinking. This underpins a consideration of the limitations of his philosophy and ontologically derived notions of weakness and passivity in imagining life with dementia as a potential site of agency or as the locus for transformative ideas about care, community, and non-instrumentalist conceptions of human value.

## Introduction: The Spectre of the Camp

The spectre of the death camp haunts a number of literary, memorial, and scholarly responses to the late twentieth and early twenty-first century global “dementia crisis.” This essay develops a critique of this analogical landscape as part of a broader endeavour to think through the ideological dimensions of the very notion of a dementia “crisis” and the ways in which this has become yoked to political, ethical, and economic evaluations of particular groups of people and to associated debates around the sustainability and management of care in later life (Burke 2016, 2017). Implicit here too is a claim about the contribution of critical and cultural theory to an understanding of contemporary cultural epistemologies of dementia. I am interested in the ways in which the kind of provocative and speculative readings that literary, theoretical, and cultural scholarship enables, unfettered by the methodological constraints of sociological or scientific enquiry, opens up a space in which to defamiliarize and trouble dominant representational modalities of dementia and assumptions about the challenges with which it presents us.

In the first part of the essay, I discuss the French feminist writer Annie Ernaux’s account of her mother’s dementia and hospitalization in *I Remain in Darkness* (Ernaux 1999). I focus in particular upon the implications of her symbolic association between the long-stay geriatric ward and the death camp wherein the person with dementia, like the condemned camp prisoner, is depicted as the abjected expression of its deathly logic. I then go on to explore the paradigm of the camp as an explanatory concept by way of a discussion of the work of the Italian philosopher Giorgio Agamben and his provocative claim in *Homo Sacer* that the camp is the “nomos of the modern” (Agamben 1998). My aim here, following scholars such as Sergei Prozorov (Prozorov 2014), is to elucidate Agamben’s philosophical account of biopolitics and the production of what he calls “bare life” in the broader context of the linguistic foundations of his thinking. Agamben’s view of the indistinct separation between the structure of language and its utterance informs his characterization of the aporetic potentiality of human experience. This indeterminacy gives rise to the potency of Agamben’s biopolitics, but it also provides a contextualization of his work that is often missing in dementia scholarship in which his work is cited, and it enables me to identify some the limitations of his thesis in relation to our thinking about dementia today. My aim is to show that these limitations are indicative of broader problems with some dominant cultural epistemologies of the “dementia crisis” and its exemplary status in current debates about personhood and care. My argument acknowledges that Agamben’s work helps us to identify the mechanisms that produce the person with dementia as one around whom key debates about value and the limits of meaningful life circulate. However, what I propose here, in the final section of the essay, is that we need to move beyond the political and ethical limitations of this paradigm in order to think differently about the political and ethical dimensions of dementia and care.

## Phantoms from Buchenwald—Reading Annie Ernaux’s I Remain in Darkness

It is difficult to imagine any other condition that elicits such forensic attention to the abjected, leaky, and shameful body than that of dementia (Burke 2016) as it is depicted in literature and lifewriting. As many scholars have noted, this symbolic economy reinforces the notion of dementia as a source of unmitigated dread or despair; a condition whose cultural legibility is routinely mediated in the gothic mode as an unfolding horror show populated by monsters and zombies (Herskovits 1995; Basting 2009; Cohen-Shalev and Marcus 2013; Chivers 2011; Behuniak 2011; Wearing 2013). However, my focus in this essay is not so much the prevalence of the gothic mode but rather the paradigm that underpins the proliferation of tropes that configure dementia as a condition between life and death. This paradigm is that of Giorgio Agamben’s concept of “bare life,” a figuration of political abjection that finds its most powerful expression in the spatial and temporal logic of the death camp. We can locate figures of “bare life” in narrative accounts of the institutionalization of people with dementia. For instance, in *I Remain in Darkness*, Annie Ernaux’s account of her mother’s final years living with dementia, she records her visits to her mother in hospital in April 1984:

Sunday 8

[…] Today she was in a different room with two bedridden ladies, both silent. She had been tied to her armchair […] I untied her to walk along the corridor and show the nurse her eyes. I so hate seeing her naked flesh when I lift her up in the hospital gown parts at the back.

In the corridor, through half -open door, I glimpsed a woman with her legs in the air. In the next bed, another woman was moaning just like one does during orgasm. Tonight everything was surreal and the sun was beating down.

Saturday 14

She is eating the strawberry tart I have brought her, picking the fruit out from the custard. “They have no regard for me here, they make me work like a slave, we’re not even fed properly.” Her perennial obsessions, her fear of poverty which I have long forgotten. Opposite us, an emaciated woman, a phantom from Buchenwald, is sitting on her bed, her back straight, a fearful expression in her eyes. She lifts up her chemise and you can see the diaper sheathing her vagina. Such scenes inspire horror on television. Here it’s different. These are women. (Ernaux 1999, 18–19)

Ernaux’s evocation of Buchenwald death camp as a means to express the “horrors” of the scene on the ward offers a deliberately provocative and disturbing image of the effects of dementia. In referring to one of the most powerful cultural touchstones of human suffering, powerlessness and abjection—the death camp of the Holocaust—this analogy crystallizes her visceral response to the sights and sounds of the hospital and the violent de-realization of the personhood of the older women she encounters. Ernaux describes her book as the unedited, transcription of notes she took during her mother’s final illness. It is thus presented to us as a form of raw, authentic testimonial that bears witness to her “bewilderment and distress” (Ernaux) in the face of her mother’s dementia. However, as Ross Chambers reminds us, the “event of witnessing” is “not so much a descriptive practice (whether historical or fictional) as it is a symbolic practice with its own distinct rhetoricity” (Chambers 2004, 35). Chamber’s point is important in that it reminds us that witnessing itself is always mediated by both the generic conventions of testimony as a form with its own distinct history, politics, and ethics (Beverley 2004; Smith and Watson 2012) and by the particular epistemological frames that make sense of that which is witnessed. In this sense, what Ernaux tells us are unedited “vestiges of pain” (Ernaux 1999, 8) cannot be viewed as either straightforward or reliable descriptions in the manner of an “eye-witness” account. Indeed, to take up and extend Kali Tal’s claim that “bearing witness is an aggressive act” (Tal 1996, 7), *I Remain in Darkness* is a text that encodes an aggression that is both outward and inward looking, an expression of extreme pain and anger. The affective power of this testimony is inextricably bound up with the emphasis on deficit and loss in dominant epistemologies of dementia.

It is notable that references to bodily abjection and matter out of place dominate the sparse journal entries that make up the narrative. These range from her mother’s “shit filled” underwear and the excrement Ernaux finds hidden in the drawer of her bedside table (Ernaux 1999) or on the floor by an armchair, to the casual exposure of her mother’s vagina and soiled diapers and the unsettling “orgasmic moaning” described in the passage above. Awash with descriptions of urine and faeces, the text thus performatively enacts and reinforces a powerful epistemology of dementia as fundamentally abject and obscene. The crucial point here is not the veracity of Ernaux’s descriptions but that they express a particular perception of dementia as inherently “obscene” and therefore suggest that these images of abjection are intrinsic to the condition itself rather than symptomatic of inadequate care, support, or understanding.

Since the Holocaust has been repeatedly configured as the most unspeakable form of suffering (Adorno 1973; Lyotard 1988), then something more significant is arguably taking place when it is evoked in narrative accounts of living with dementia. Indeed, the symbolic power of this analogy resides in its extreme encapsulation of the perceived impact of dementia upon personhood. Its enunciation enacts the near absolute dissolution of the unique, concrete, and particular features of the individual into a generic category of suffering marked by a type of death in life, thus reducing dementia to an expression of the abyssal point of humanity and the terminus of hope.

The symbolic association between the institutionalized person with dementia and the death camp encapsulated in the reference to Buchenwald is not peculiar to Ernaux. Phantoms from the Holocaust haunt many cultural, journalistic, and theoretical representations of dementia, aging, and institutional care in more or less explicit ways (Goldman 2017). For example, we can see precisely this association in Catherine Malabou’s philosophical account of the psychical trauma enacted by Alzheimer’s disease (Malabou 2012). Malabou’s discussion of dementia is underpinned by her elaboration of Bruno Bettleheim’s now largely discredited work on the similarity between children with autism and the *Musselmann* (Levi 1989) of the death camps (Bettelheim 1972). Malabou develops Bettleheim’s comparison between the traumatic symptoms manifest by people in the camps and neurological damage in order to describe the effects of Alzheimer’s disease. Combining Bettleheim’s impressionistic assertions with selected neuroscientific claims culled from the work of Antonio Damasio (1999, 2003, 2006), Joseph LeDoux (2002), and Oliver Sacks (1973, 1995, 1998), she thus develops her hypothesis that “patients with Alzheimer’s disease or, more generally, patients with brain lesions, behave as if they are suffering from war trauma” of the kind associated with Holocaust survivors (Malabou 2012, xviii). In keeping with this claim, her description of her grandmother’s transformation because of Alzheimer’s into “the work of the disease, its opus, its own sculpture” (xi) is symbolically aligned with Ernaux’s reference to the “phantom from Buchenwald” (Burke 2017, 89). Both writers emphasize the evacuation of the person in the face of the organic destruction wrought by the disease.

With this sense of extreme and absolute nature of suffering in mind, it is important to note that the association between the death camp and the long-stay ward or care home often reflects more than symbolic correspondences between the two spaces (Capstick 2017; Leibing 2006; Goldman 2017, 220). References to the similarity between the camp “victim” and the person with dementia occur in newspaper reports on poor care as a means to express something of the violence of institutional imprisonment and neglect. For instance, in 2008 the U.K. tabloid, *The Daily Mail* ran a headline “Care home left my mother looking like a concentration camp victim” (Hale 2008), and in December 2016, a number of newspapers in the United Kingdom reported the case of a Holocaust survivor in her nineties who compared her experience in a care home to being imprisoned in a Nazi camp (see, for example, Bingham 2016).

In *I Remain in Darkness*, Ernaux notes that on the geriatric ward her mother must wear garments provided by the hospital and “can have nothing of her own” (30). If this recalls the removal of the clothing and possessions of the camp prisoners then the condition of the hospital wards, reeking of “food, urine and shit” and the habitual use of restraints to tie unruly patients down describe an environment that reduces people to an animal existence; “The place is a cage. My mother is a solitary figure” (30).

This sense of profound spatial isolation is coupled with an emphasis upon temporal dislocation and the separation of the person from the rhythms and rituals of social life. As Ernaux notes, the space of the ward is one in which the passage of chronological time no longer matters:

Wednesday 5

Indoors, the same warm temperature, year-round. There are no more seasons. (32)

[…]

Sunday 9

There are clocks all over the place, in the hall, the dining room and the bedrooms; none of them gives the right time: 6 o’clock instead of 4 o’clock … Do they do it on purpose? (42)

This description of life on the ward is of an existence outside meaningful temporal divisions, in which conventional markers of progress and change are suspended; there are “no more seasons” and none of the clocks tell the right time. This aligns the temporal dimensions of residential care with Giorgio Agamben’s highly influential vision of the camp as a liminal “zone of indistinction” (Agamben 1998, 25), a place in which people exist in a state of exception, stripped of their legal and political identities in a time and space between life and death. We see another example of this in the following passage:

On New Year’s Day my mother and the other patients had been dressed in their former clothes … A parody of real life … The women seem to be vaguely waiting.

There is nothing to wait for … Here it is all in the past; there are no more real parties to look forward to … (45)

Existing in an absurdist Beckettian universe vaguely waiting for nothing, Ernaux’s description of the patients here re-inscribes the culturally dominant perception of dementia as a form of “biosocial death” (Leibing 2006) or a “death in slow motion” as Eleanor Cooney puts it (Cooney 2003). The long-stay geriatric unit is presented as a space in which meaningful life and social inclusion come to an end in the period leading up to physical death. Ernaux emphasizes an indifference that bespeaks the devaluing of life; “here, the things that get lost are never found. No one cares: they’re going to die anyway” (30).

There are many very suggestive and illuminating analyses of aging and care in Ernaux’s work and of the literary rendering of her complex and often troubled relationship with her mother (Motte 1995; Miller 1999; Jordan 2011; Zimmermann 2017). However, what I want to draw attention to here are some of the ethical implications of the analogical mediation of dementia via Holocaust metaphors and the correspondences between the aged care facility and the spatiotemporal characteristics of the concentration camp. As I noted above, the symbolic power of this analogy, and perhaps the reason for its use, resides in its extreme encapsulation of the perceived impact of dementia upon personhood. However, this symbolic power is, I would argue, problematic for a number of reasons. In the first instance, the analogy used by the writer/observer symbolically reinscribes the very impersonal and debased effects that the self-same writer or observer laments as attributes of the neurological impairments associated with dementia. The peculiar, distinctive subject at the performative centre of life-writing as an ethical practice (Eakin 2004, 4) is symbolically displaced. Instead, the intelligibility or meaning of dementia as a lived experience is communicated via a broader, de-individuated cultural category—the death camp “victim”—an ontology that is defined only in terms of suffering, deficit, horror, loss, vulnerability. What we have here then is a representational modality that effaces the contours of the person in order to communicate something about the emotional impact of dementia on the writer/observer. This is explicit in Malabou’s description of her grandmother as:

… a stranger who didn’t recognise me, who didn’t recognise herself because she had undoubtedly never met her before. Behind the familiar halo of hair, the tone of her voice, the blue of her eyes: the absolutely incontestable presence of someone else. (Malabou 2012, xii)

In this description, Malabou’s grandmother becomes the “absent centre” around which she elaborates her discussion of cerebral trauma and her own emotional suffering. In a similar manner to Ernaux’s description of the silent woman with whom her mother shares a room, these descriptions construe the lived experience of dementia as opaque and unrepresentable, characterized by a fundamental rupture of intersubjectivity in that the gaze of the observer is never returned or acknowledged. This paradigm precludes recognition of the diverse ways in which individuals might live with particular impairments as well as any notion of the person with dementia as someone capable of meaningful interaction, of expressing a preference or exercising choice or agency. I will return to this distinction between a life with dementia and, what we might call, a death-in-life due to dementia later in my analysis of the different theoretical paradigms in contemporary theory that sustain these ideas, notably in the work of Giorgio Agamben.

The second problem with the death camp analogy is that it is an abstraction. The postulation of a figure of universal suffering (the camp victim) serves to extract the person with dementia from the concrete particularities of lived experience and dislocate them from the specific determinants of history. It is remarkable, for instance, that Ernaux does not connect her mother’s disposition, distress, or behaviour on the hospital ward to the standards and management of her care. It is as if there is no relationship between aspects of her mother’s decline and inhabiting an environment in which people are tied to chairs, surrounded by piles of faeces and the smell of stale, sticky urine. That the significance of her environment is not addressed arguably tells us a great deal about the explanatory power accorded to Alzheimer’s disease as a diagnostic category as if all that Ernaux is able to “see” and record follows from the pathological process of the disease itself. However, it is difficult not to make these connections in any reading of the text attentive to the repetition of references to bodily matter, the exposure of flesh, diapers, and underwear and the reported indifference and casual brutalities of the care regime.

## From Literature to Cultural Critique

The spectre of the death camp also haunts critical scholarship on dementia in the Arts, Humanities, and Social Sciences. For instance, Annette Leibing, Andrea Capstick, and Sadie Wearing amongst other scholars in this field, make reference to Giorgio Agamben’s concept of “bare life” in their discussions of the legal and ethical status of people with dementia, particularly in relation to end-of-life care in residential settings (see Leibing 2006; Capstick 2017; Wearing 2013). For Agamben, we are living at the “catastrophic endpoint of a political tradition that originates in Greek antiquity and leads to the National Socialist concentration camps” (Lemke 2005, 4). His concept of bare life which he describes as the “fundamental activity of sovereign power” (181) unfolds around his development of Carl Schmitt’s claim that there is an “essential contiguity between the state of exception and sovereignty” (Agamben 2005, 1). Schmitt’s definition of the sovereign—“he who decides on the state of exception”—revolves around the paradoxical “fact that the sovereign is, at the same time, outside and inside the juridical order” (Agamben 2005, 2). The sovereign is both inside the law because the juridical field is coterminous with his territorial domain, yet also outside the law due to his ability to suspend the legal order and declare a state of emergency.

Noting the ambiguity that surrounds the definition of sovereign power as the power to “decide on the state of exception,” Agamben draws attention to the implications that follow from the power to enact juridical measures to suspend the law itself. He uses the laws enacted by the Nazi regime first to suspend articles of the Weimar Constitution and then to strip Jewish people of their citizenship as an example of this phenomenon—what he describes as the “voluntary creation of a permanent state of emergency” (2005, 2). The state of exception thus “appears as the legal form of what cannot have legal form” (2005, 1).

Agamben locates the origins of this constitutive ambiguity in the Aristotelian distinction between biological life (*zoé*) and political existence (*bíos*) or between “law and the living being” (Agamben 1998, 2). Sovereign power is thus manifest in the articulation of a threshold between legal-political status and “bare life.” It is important that we understand the latter as a politicized category rather than as biological existence per se; “bare life” is an attribution predicated upon the exclusionary logic of the state of exception. It is produced rather than revealed by the operative mechanisms of sovereign power. This is exemplified for Agamben in the figure of *homo sacer* derived from Roman Law (1998). This sacred man denotes the human who can be killed without the commission of a crime, in other words, one who is no longer subject to the rights and recognition conferred by legal or political status (1998). As he puts it, “every society sets this limit; every society—even the most modern—decides who its ‘sacred men’ will be” (1998, 139). Thus the stripping of citizenship from Jewish people by the Third Reich facilitated the genocide; the concentration camp prisoners could be killed without the commission of a criminal act. However, whilst the production of “bare life” is, for Agamben, a longstanding characteristic of sovereign power, he argues that today the “state of exception tends increasingly to appear as the dominant paradigm of government in contemporary politics” (2005, 2), producing a “threshold of indeterminancy” between democracy and totalitarianism (1998, 121) or absolutism (2005, 2). It also means that “we are all virtually *homines sacri*” (1998, 115):

Along with the emergence of biopolitics, we can observe a displacement and gradual expansion beyond the limits of the decision on bare life, in the state of exception, in which sovereignty consisted. If there is a line in every modern state marking the point at which the decision on life becomes a decision on death, and biopolitics can turn into thanatopolitics, this line no longer appears today as a stable border dividing two clearly distinct zones. This line is now in motion and gradually moving into areas other than that of political life, areas in which the sovereign is entering into an ever more intimate symbiosis not only with the jurist but also with the doctor, the scientist, the expert, and the priest […] From this perspective, the camp—as the pure, absolute, and impassable biopolitical space (in so far as it is founded solely on the state of exception)—will appear as the hidden paradigm of the political space of modernity. (1998, 122–123, my ellipses)

In developing his thesis, Agamben refers to distinctively modern categories such as “overcoma” and “brain death” (1998, 160–165) as examples of the attribution of human lives that can be killed without the commission of a crime and of the enmeshing of political power with the institutions and authority of medicine and the law. As this indicates, his argument is that the thanatopolitical logic of contemporary power is not simply manifest in the genocidal intent of totalitarian regimes but encompasses us all, finding its expression in multiple domains from the internment of “non-lawful enemy combatants” in Guantanamo Bay (see Gregory 2004) to the decision to terminate life support or legally to assist suicide.

## Bare Life and the Case of Peter Singer

We can see Agamben’s “hidden paradigm” reflected in the preference utilitarianism of bioethicists such as Peter Singer. Singer’s call to “rethink life and death” in the context of the capacity of contemporary medical technologies (Singer 1996) to sustain, terminate, and produce new forms of life is predicated upon a notion of the differential value of some lives over others. Singer’s argument is that we must separate personhood from species being and that personhood itself should be established with reference to the Lockean concept of psychological continuity and the capacity to understand oneself as a being in time (with a past and a future), allied notions of autonomy, the capacity to plan and to desire and to value being alive. Medical and legal expertise play a key role in the determination—or perhaps more accurately—the attribution of personhood in Singer’s work. Like Agamben’s sacred men, Singer’s non-persons are produced by the articulation of a distinction between the person and the merely human. Significantly for any discussion of dementia, in a section of *Rethinking Life and Death* on voluntary euthanasia, he presents the case of Janet Adkins, a woman in the early stages of Alzheimer’s who decided to end her life with the help of Jack Kevorkian before her condition progressed to the extent that she was unable to make such a decision. He notes:

Adkins tried an experimental treatment for Alzheimer’s. It didn’t work. She then decided that she wanted to die, while she was still capable of thinking coherently. She had heard of a Michigan pathologist, Dr Jack Kevorkian, who had developed a “suicide machine” and offered to make it available to people who were incurably ill and wanted to die … She contacted Dr Kevorkian who discussed her desire to die with her, and then asked her to get her doctor to send him her medical records. After he had studied them, Kevorkian agreed to help Adkins. (Singer 1996, 133)

Devoid of any exploration of family dynamics, the particular people and personalities involved, and of the potentially overwhelming emotional freight that must have accompanied this decision, Singer’s version of the case presents Adkin’s decision as both an exemplar and justification of what he presents as an emergent ethics of choice in the domain of life/death decision-making. It is evoked to add further ballast to his wider argument that the traditional ethical principle of the sanctity of human life should be displaced in favour of a judgement based upon “choice” (i.e. making a decision to die) or a test of personhood. His narrative concludes with reference to the five medical members of the jury that eventually tried and acquitted Jack Kevorkian (over the case of Thomas Hyde, a man with Lou Gehrig’s disease, rather than Janet Adkins), two of whom are cited as evoking an individual’s “right” to choose whether to live or die (1996). Of course, the very evocation of a discourse of “rights” in this context underlines the extent to which the notion of choice as a right is conceptualized in legal terms and thus not a matter of an unmediated individual freedom “to choose” at all.

Singer retains a strong sense that it remains in the pay of legal and medical experts to validate and to confer such a right to choose upon the individual. His account makes it clear that it is Kevorkian who actually makes the decision to facilitate Adkins’ suicide after the scrutiny of her medical records, and it is the medical professionals on the jury who validate Kevorkian’s actions and thus implicitly support Singer’s argument. He constructs the plight of Adkins and those in similar positions primarily as a problem for “jurists and doctors” (see Agamben 1998, 136–143) and ethicists such as himself rather than for those personally involved in the particular dilemma at stake.

The coalescence of medical and politico-legal elements in Singer’s work exemplifies Agamben’s claim that today “the physician and the scientist move in the no-man’s-land into which at one point the sovereign alone could penetrate” (1998, 159). The claim that today “bare life inheres within us all” (1998, 115) is certainly integral to the re-envisioning of life, death, and personhood we see in Singer’s work and in broader debates around capacity, cognition, and personhood and euthanasia and assisted dying. Crucially, for Agamben, the politico-legal and medical nexus that underpins the definition of personhood and decision- making around the end of life is wholly continuous with the logic of the concentration camp:

…if the essence of the camp consists in the materialization of the state of exception and in the subsequent creation of a space in which bare life and the juridical rule enter into a threshold of indistinction then we must admit that we find our self virtually in the presence of a camp every time such a structure is created, independent of the kinds of crime that are committed there. (1998 174)

Viewed through the lens of Agamben’s philosophy, Singer’s situation of a criterialist concept of personhood within a medical and legal framework exemplifies the operative mechanisms of the state of exception and Agamben’s notion of “bare life” as an attribution. Whilst both philosophers view the politicization of life itself from very different ethical perspectives, they both endorse a fundamentally legalistic conception of biopolitical governmentality (Lemke 2005), at the centre of which is a notion of an essentially powerless human subject whose legal and political recognition is not a given. It is not difficult to see why Agamben’s concept of “bare life” has been appropriated in scholarship that explores the notion of dementia as a form of “biosocial death” (Leibing 2006) and the residential care home as death camp (Capstick 2017); a liminal space in which rights and recognition are suspended. We can certainly identify affinities between Agamben’s philosophical discourse and the figurative language and tropes that pervade Ernaux’s writing. Agamben’s crystallization of the mechanisms of contemporary biopolitics in the paradigm of the camp is a powerful and compelling symbol of contemporary sovereign power and the capacity of the state to remove particular groups of people from the protective framework of legal, political, and social recognition. However, in a similar manner to Ernaux, his work offers up an essentially abstract envisioning of the powerless and abjected human subject “materialized” by the exclusionary logic of the state of exception. Although his thesis makes reference to a historical event—the Holocaust—it is profoundly ahistorical in its utilization of the camp as a paradigmatic structure rather than a distinct historical occurrence.

We may locate some of the reasons for this ahistoricism in the linguistic rather than historical/political underpinnings of his theory. Understanding the linguistic foundations of Agamben’s thinking is important if we are to grasp the ethical and political implications of his work and its usefulness in relation to our understanding of the political and ethical dimensions of thinking about dementia today. It is to these foundations that I wish now to turn.

## Language, Law, and Life

In the introduction to their collection of essays entitled, *The Work of Giorgio Agamben*, the editors, Justin Clemens, Nicholas Heron, and Alex Murray draw attention to the specifically poetic and linguistic foundations of Agamben’s thinking. Emphasizing the continuity between his earlier writings on the philosophy of language, aesthetics, and literature and his later more explicitly political engagement with questions of biopower and sovereignty, the collection is subtitled “Law, Literature, Life.” In his contribution to the collection, “The Role of the Shifter and the Problem of Reference in Giorgio Agamben,” Justin Clemens argues that Agamben’s fundamental ontological concerns with the category of potentiality and the typological paradox of the state of exception, cannot be separated from the conceptual problems related to deixis or the linguistic shifter. “If one misses the centrality of Agamben’s philological deconstruction of grammatical problems to his thinking as a whole,” Clemens notes, “one will also miss what’s important about his method and key concepts” (Clemens 2008, 47). Indeed, “the problem of being—the supreme metaphysical problem,” as Agamben himself argues, “emerges from the very beginning as inseparable from the problem of the significance of the demonstrative pronoun” (Agamben 1991, 16–17).

Demonstrative pronouns are pronouns that identity specific nouns, for example, this, that, these, and those. They are examples of the grammatical category known as deixis, also described as “shifters.” This category, often described as “verbal pointing,” refers to expressions and markers in language that cannot be fully comprehended without additional contextual information relating to person (“I,” “you”), place (“here,” “there,” “this,” “that), time (“now,” “then”). For instance, the utterance “this city” is only meaningful if we are aware of the city in which the utterance takes place. Deixis thus entails the suturing of the utterance to the speaker and the moment of enunciation (Clemens 2008, 46). It is the particular characteristics of these shifters that interest Agamben:

It is certainly possible to define something like a meaning of the shifters “I”, “you”, “now”, “here” but this meaning is completely foreign to the meaning of other linguistic signs. “I” is neither a notion or a substance, and enunciation concerns not what is said in discourse but the pure fact that it is said, the event of language as such, which is by definition ephemeral. Like the philosophers’ concept of Being, enunciation is what is most unique and concrete, since it refers to the absolutely singular and unrepeatable event of discourse in act; but at the same time, it is what is most vacuous and generic, since it is always repeated without it ever being possible to assign it any lexical reality. (Agamben 1999b, 138)

As Clemens (2008) notes, Agamben’s argument here draws upon Emile Benveniste’s theorization of the shifter as that which enacts the deconstruction of the grammatical dualism that founds much of modern linguistics, namely the distinction between what Ferdinand de Saussure terms *langue* (the structure of language or relations between signs in a language system) and *parole* (the individual speech utterance) (Saussure 1983; Benveniste 1973). On the one hand, a pronoun such as I is conventionally accepted to represent an “individual concept”’ or element within *langue*. On the other hand, since the I can be assumed by various different speaking subjects in a linguistic utterance, “there is,” argues Benveniste “no, concept of I encompassing all the I’s uttered at every single moment by every single speaker” (1973, 226). In other words, the “I” has no positive relation to an individual concept within langue: “there is no lexical entity named by the I” (226). However, the situational nature of the shifter within linguistic discourse—the fact that it refers to an immediate context of utterance that is changeable—also means that a pronoun such as “I” cannot be located simply on the plane of the utterance or parole. As Benveniste concludes, the personal pronoun “can only be identified by the instance of discourse” or the “act of speaking in which it is uttered” (218). The significance of the shifter or personal pronoun therefore refers to an extra-conceptual and pre-subjective “reality of discourse” that can be conceived of as the taking place of language. Importantly, this taking place deconstructs the dualistic distinction between *langue* and *parole* (linguistic structure and utterance) because personal pronouns, as Benveniste notes, “are distinguished from all other designations a language articulates in that *they do not refer either to a concept or to an individual*” (226).

In terms of Agamben’s philosophy, what we see in his consideration of the shifter is an account of the elision of two previously distinct categories—*langue* and *parole*—in a zone of indistinction. The “I” is both “inside and outside the categorical” and both “unique and vacuous” (Attell 2015, 68). The shifter has no real material or conceptual referent apart from the fact of its taking place. It is thus, “the transcategorical ontological operator that effects the link between the linguistic and the extra linguistic” (Attell 2015, 66). Crucially, for Agamben, the shifter’s transitional nature is related to a profound ontological destiny of human beings. As he notes in *Infancy and History*, the distinction between humans and other living animals is not simply produced via a notion of linguistic ability alone. Animals, he argues “are already inside language” whereas humans precede speech in a state of “infancy” and “in order to speak [man] has to constitute himself as the subject of language—he has to say ‘I’” (Agamben 1993, 59–60). The difference between a human animal and a human being as such is, then, marked by this destinal transition between language and discourse that takes place as the human emerges from their infancy. According to Agamben, however, this “reality of discourse”—the transition between *langue* and *parole*—theoretically structures every instance of human language and thus the human being is said to exist in a permanent state of infancy, as he terms it. As such, human life is configured as a bare space of potentiality that is marked by a distinct elision of two previously distinct concepts that enter into a state of exception or indistinction. The human is thus, according to Agamben, a “whatever being” (Agamben 2005), defined by “nothing other than this very passage from pure language to discourse” (Agamben 1993, 64).

Agamben thus develops a critical system that typically operates by revealing the elision between the dualistic divisions of Western metaphysics. His ontological theory of human potentiality is, like his concept of bare life, achieved via an operational mechanics that brings together two previously distinct categories into aporetic zone of indistinction or state of exception. Indeed, as Antonio Negri notes, for Agamben, the logic of biopolitics and human infancy means that all aspects of human sociality are “bipolar and transitive: home and city, zoe and bios, life and politics, [*langue* and *parole*], flow from one to the other, and are situated within an ever reversible flow” (Negri 2007, 117).

## Conclusion: Beyond the Paradigm of the Camp

Acknowledging the linguistic foundations upon which Agamben’s thinking is predicated is vital if we are fully to understand the provenance of his writings on history and contemporary biopolitics (Prozorov 2014) It also enables us to reflect critically on the political and ethical limitations of figurations of dementia as a form of “bare life” and the aligned criterialist concept of personhood that we find in Peter Singer’s work. (The latter concept, as I indicate above, enacts the form of biopolitical governmentality that Agamben describes).

When placed within this broader linguistic and ontological framework, Agamben’s concepts of *homo sacer* and bare life can be viewed as metaphors for his linguistic ontology; evocative figures of speech but ultimately ones that can be severed from the particular historical situations in which they may be deployed as explanatory or “meaning-making” devices. Although Agamben’s philosophy bears the scars of history, particularly that of the Holocaust death  camp, the notion of “bare life” is essentially ahistorical—the expression of a philosophical legacy rather than of the concrete particularities of history itself. Thus, as many scholars have noted, the powerful claim that bare life inheres within us all (Agamben 1998) fails to differentiate between radically distinct scenarios, for instance, between the person on life support with a cataclysmic brain injury and the prisoners in the camp (see Rabinow and Rose 2006). Nor is it able to address the specific historical and economic determinants that render people more or less vulnerable to this attribution.

We can therefore see how the ahistorical nature of Agamben’s concept of bare life lends itself to a medical epistemology of dementia that foregrounds particular pathological processes and symptoms at the expense of the peculiarities and context-specific experiences of living with dementia. When the rich, messiness of embodied experience is reduced to the stark contours of a diagnosis we place the historicity of dementia itself in parenthesis and assume instead a stable and knowable “disease” process without “history.” The effects of this are manifest in the emphasis upon abjection and passivity that we see in Ernaux and Malabou’s descriptions of dementia as both the origin of suffering and of the destruction of personhood as if the peculiarities of personal history, relationships, environment, culture, and community have no bearing on their perception.

As I have indicated above, it is no surprise then that these cultural configurations of dementia lend themselves to a theoretical engagement with the work of Agamben. As one of Agamben’s main interlocutors, Alain Badiou puts it, Agamben envisions the human as essentially delicate or “crushed by the crass commotion of powers” (Badiou 2009, 558–559). Whilst this has obvious descriptive and empirical force, there are, as Badiou also suggests, potential problems with the postulation of bare life as our ontological or linguistic destiny and I would argue that these problems are particularly acute for scholarship on dementia that attempts to think about the ethics and politics of long life, illness, dependency, and care.

Agamben’s configuration of our ontology is one that foregrounds the weakness or passivity of humans as destiny. Our abjection, whether potential or real, is something that inheres within us all. For Badiou, this is problematic for it is grounded upon an ahistorical conception of humanity envisioned as an essentially passive figure reduced to a state of pure being. Rather than enquire into the possibilities for what Badiou calls the “affirmative becoming of truths” (Badiou 2009, 514) or historical and social change, the very nature of Agamben’s ahistorical linguistic ontology precludes the concrete realization of a new vision of human community. The concept of bare life in this sense reinscribes the vulnerability it describes rather than offering a way of thinking beyond it.

Comparisons between the care home and the camp and between the person with dementia and the Holocaust victim serve as powerful expressions of abjection and vulnerability at the limits of life. However, these figurations, based as they are on a linguistic and thus ahistorical ontology, ultimately occlude the historicity of particular ways of managing and organizing care, and of understanding cognitive impairment in later life. Instead, the diverse ways in which people live with dementia are subsumed within a powerful symbolic abstraction that precludes us from imagining life with dementia as a potential site of agency or as the locus for transformative ideas about care, community, and non-instrumentalist conceptions of human value. With this in mind, I would propose that any ethically based engagement from scholars that intends to identify and also transform the current constellation of social, institutional, economic, and medical practices that determine our understandings and experience of dementia must attend to the implications of the theory on which they ground this very engagement. As I have argued above, a theory grounded on ontologically derived notions of weakness, passivity, and remainders may not be what we are looking for or what we need. Instead, in our work may want to think about new foundations for our understanding of living and experiencing old age, dependency, and dementia.
